# Melanism influences the use of social information in a polymorphic owl

**DOI:** 10.1038/s41598-020-58826-6

**Published:** 2020-02-05

**Authors:** Deseada Parejo, Jesús M. Avilés

**Affiliations:** 1 Área de Zoología, Departamento de Anatomía, Biología Celular y Zoología, Facultad de Ciencias, Universidad de Extremadura, Badajoz, Spain; 2 Departamento de Ecología Funcional y Evolutiva, Estación Experimental de Zonas Áridas (EEZA-CSIC), Almería, Spain

**Keywords:** Behavioural ecology, Evolutionary ecology

## Abstract

Social information use has well-known fitness benefits. However, causes underlying the apparent inter-individual variability in the propensity to use social information are poorly studied. Melanins are pigments responsible for most of intra-specific color variation in vertebrates and their variation is often associated with changes in behaviour. Here, we explored whether melanism is related to individual propensity to use social information in the color polymorphic scops owl *Otus scops*. We manipulated social information on predation risk at nests by broadcasting calls of the sympatric little owl *Athene noctua* and found that owlets of brownish females exposed to alarm calls had lower levels of natural antibodies than those of greyish females. In parallel, we found changes in parental behaviour contingent on coloration because when exposed to the risky treatment brownish females returned earlier to nests than greyish females and owlets raised by brownish females were fed with smaller prey than those raised by greyish ones. These results provide support for a previous ignored role of melanins on the propensity to use social information, which may help to explain the maintenance of melanin-based color polymorphisms wherever social environments are variable.

## Introduction

The use of social information is a common and effective mechanism by which animals improve their assessment of the quality of resources^1^. Its use has been demonstrated in contexts as diverse as mate choice e.g.^2,3^, predator avoidance e.g.^4,5^ and foraging and breeding habitat selection e.g.^6–8^, having clear ecological and evolutionary consequences^9–13^. Empirical studies have reported the use of social information by different species from very distant taxa as fishes e.g.^7^, insects e.g.^2,14^, amphibians e.g.^15^, birds e.g.^4–6^ and mammals e.g.^16,17^. However, considerably far less attention has been paid to intra-specific variation in the propensity to use social information, despite accumulated evidence showing that is not a species-specific characteristic^18–20^. In particular, the mechanistic basis behind such intra-specific variability remains poorly understood^21,22^. This is unfortunate given the important ecological and evolutionary role of behavioural variation among individuals^23^.

Here, we proposed a mechanism that could help explaining intra-specific variability in the propensity to use social information within populations. In vertebrates, melanin-coloration is often linked to variation in morphological, physiological, behavioural and life-history traits^24–33^, potentially leading to the integration of these traits into complex melanic syndromes over which selection may act^34^. This raises expectations that melanin-based coloration might be related to social information use. Many studies have shown an association between melanism and behaviours such as sexual activity, aggressiveness, boldness, exploration and sociability in vertebrates (see reviews in^24,34,35^). For example, darker males have shown to be sexually more active, more aggressive toward conspecifics and more dominant than lighter males from the same population. Moreover, some of these behaviours like boldness^18,36^, exploration^37,38^ and sociability^30^ have been related to inter-individual differences in the degree of social information use in several fishes and birds. Most of these studies focused on eumelanic coloration, but there is also some evidence supporting the occurrence of the covariation between phaeomelanic coloration and behaviour. For instance, in the polymorphic barn owl (*Tyto alba*), more phaeomelanic individuals showed the most prosocial phenotype, suggesting a link between phaeomelanins and social behaviour^39^. Therefore, behavioural differences related to melanin-based, either eu- or phaeo-melanin, coloration might determine the propensity of individuals to use social information.

In this manuscript we test for the first time whether melanin-based coloration is linked to the propensity to use social information in a color polymorphic bird, the Eurasian scops owl (*Otus scops*) (scops owl hereafter). For that purpose, we manipulated social information on predation risk by means of calls of the sympatric little owl (*Athene noctua*) near nests and measured the response in terms of parental fitness (number, weight and immunity of owlets at the nests) and behaviour (nest attendance and food provisioning) of individuals differing in the extent of melanism (brownish *versus* greyish individuals). In our scops owl’s population plumage redness variation is due to phaeomelanin, so that brownish individuals show higher values of phaeomelanin on the head and breast feathers than greyish ones^40^. Also, in our population, phaeomelanic plumage covaries with boldness in adult birds (Cruz-Miralles, Avilés, Chastel, Expósito-Granados & Parejo, unpublished). This covariation between melanism and boldness could be extensive to sociability and, hence, to the tendency to use social information, which is what we want to test here. This system is ideal to test for a role of melanism on social information use given previous evidence demonstrating that scops owl rely on social information provided by the calls of little owls when selecting nest-sites^5,41^. Alarm calls constitute a source of social information telling about the type and intensity of threats^42^. During the breeding period, information about predation risk might lead to different behavioural strategies to cope with the level of threat, and this could affect fitness prospects^43^. Therefore, we looked for an effect of the manipulation of social information on parental fitness in relation to melanism. As commonly used proxies of parental fitness we used brood size at fledging e.g.^44^ and mean fledging weight per brood e.g.^45^. In addition, we measured levels of Natural Antibodies (Nabs hereafter) in fledglings as a component of innate immunity that has been positively related to fecundity^46^ and survival^47,48^ in other bird species. In a second step, we tested whether the experiment leaded to changes in parental behaviour in the short-term that could help to understand the potential effects on fitness.

## Results

### Effects of the social information treatment on parental fitness

**Figure 1:**
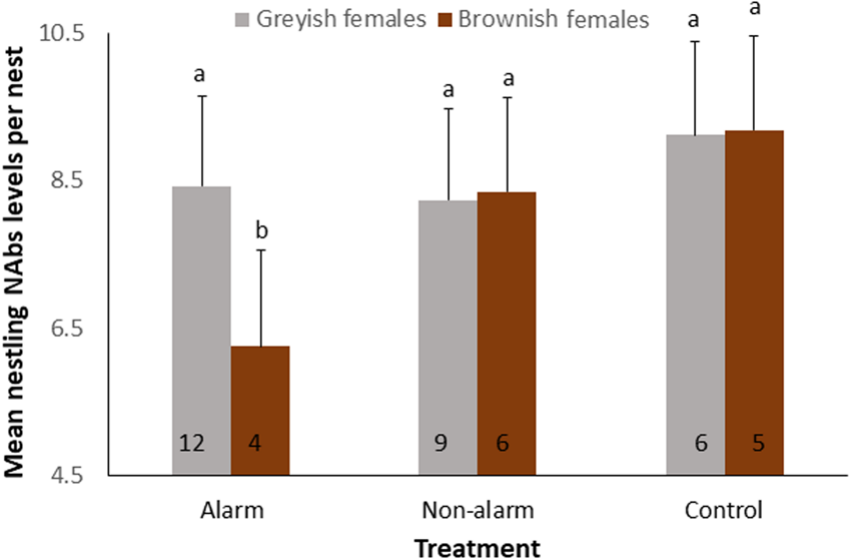
Immunology of nestlings under the different treatments. Differences in mean (+se) values of NAbs level of nestlings per nest in relation to the social information treatment (alarm nests, n = 16; non-alarm nests, n = 15; control nests, n = 11) for nests raised by brownish (brown columns) and greyish (grey columns) females. High levels of NAbs indicate good immunity. Number of nests within each group are shown inside columns. Letters above columns indicate statistically significant different groups.

Neither brood size nor mean weight of owlets per brood at fledging were affected by the social information treatment (calls exposure), neither as additive effect nor in interaction with female color (Table 1). However, NAbs levels in owlets were related to the interaction between the social information treatment and the female color (Table 1): owlets of brownish females in alarm nests, but not in non-alarm or control nests, showed lower levels of NAbs than those of greyish females (Fig. 1, Tables S1, S2).

**Table 1:** Results of General lineal models testing for the effect of the experimental treatment and the female color on the number of fledglings, mean weight of fledglings and mean NAbs levels in fledglings per nest.

	Number of fledglings			Mean fledgling weight			Mean Nabs level in fledglings		
	n = 43 nests			n = 40 nests			n = 42 nests		
	DF	χ	p	DF	F	p	DF	F	p
Intercept	1	**6.67**	**0.01**	**31**	**5.08**	**<0.001**	**34**	**12.49**	**<0.001**
Treatment	2	1.49	0.47	2,31	0.53	0.59	**2,34**	**7.13**	**0.003**
Female color	1	1.43	0.23	1,31	0.19	0.67	1,34	2.84	0.10
Treatment*female color	2	1.42	0.49	2,31	1.62	0.21	**2,34**	**3.97**	**0.03**
Laying date	1	**4.06**	**0.04**	**1,31**	**5.61**	**0.02**	1,33	0.24	0.63
Year	2	2.95	0.23	**2,31**	**5.78**	**0.01**	**2,34**	**43.91**	**<0.0001**

Laying date, which was included as a covariate in all models, was backward removed from the models when it was far from significance (P > 0.1). Significant terms of the final model are highlighted in bold. Degrees of freedom for fixed effects in models with Normal error distribution were estimated using the Kenward-Roger approximation.

### Effects of the social information treatment on parental care

**Figure 2:**
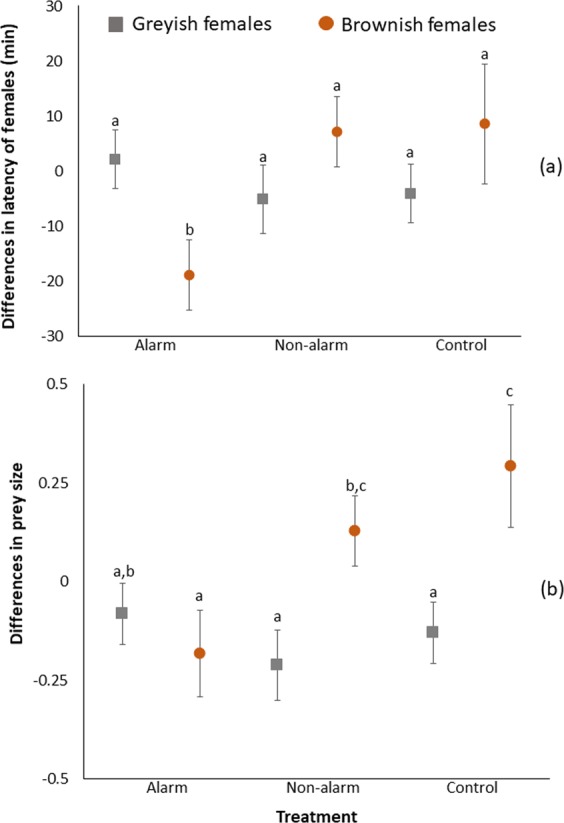
Differences between the pre- and during- treatment (alarm nests, n = 7; non-alarm nests, n = 6; control nests, n = 5) periods in: **(a)** latency of females (mean ± se) to resume usual activities at nests for brownish (brown dots) and greyish (grey squares) females; and in **(b)** relative prey size (mean ± se) brought at each nest by the two parents, for nests raised by brownish (brown dots) and greyish (grey squares) females. Letters above bars indicate statistically significant different groups.

Latency to resume usual female activity at the nests was differently affected by the social information treatment (calls exposure) in brownish and greyish females (Table 2). Brownish females exposed to the alarm treatment decreased the time to resume their usual activities in the during-treatment compared to the pre-treatment time (Fig. 2a, Table S3). Meanwhile, greyish females responded similarly to all social information treatments (Alarm calls, Non-alarm calls and Control) (Fig. 2a, Table S3). Neither change in latency of males, nor in provisioning rates of any of the sexes were affected by the social information treatment, either alone or in interaction with the parental color (Table 2). However, the change in mean prey size from the pre-treatment to the during-treatment time was affected by the social information treatment in interaction with the female but not with the male color (Table 2). In nests raised by brownish females, prey size did not differ between the pre- and the during-treatment periods in the alarm treatment, but it increased in nests under the non-alarm and control treatments (Fig. 2b, Table S3). Meanwhile, prey size did not change between periods in nests raised by greyish females (Fig. 2b, Table S3).

**Table 2:** Results of General lineal models testing the effect of the experimental manipulation and individual color on differences between pre- and treatment time in latency of males and females to resume usual activities, in provisioning rate of male and females and in mean size of provisioned prey per nest.

	Females						Both adults		
	Latency			Provisioning rate			Mean prey size		
	n = 18 nests			n = 18 nests			n = 17 nests		
	DF	F/*t*	P	DF	F/*t*	p	DF	F/*t*	p
Intercept	**11**	**4.35**	**0.001**	12	0.30	0.76	11	1.88	0.09
Treatment	2,11	1.64	0.24	2,12	0.66	0.53	2,11	1.89	0.20
Female color	1,11	0.05	0.82	1,12	0.39	0.55	**1,11**	**6.84**	**0.02**
Treatment*female color	**2,11**	**4.89**	**0.03**	2,12	1.28	0.31	**2,11**	**3.89**	**0.05**
Laying date	**1,11**	**16.35**	**0.002**	1,11	1.05	0.33	1,10	0.48	0.50
	**Males**						**Both adults**		
	**Latency**			**Provisioning rate**			**Mean prey size**		
	**n = 15 nests**			**n = 15 nests**			**n = 14 nests**		
	**DF**	**F/*t***	**P**	**DF**	**F/*t***	**p**	**DF**	**F/*t***	**p**
Intercept	**9**	**2.49**	**0.03**	**8**	**2.25**	**0.05**	8	0.34	0.74
Treatment	2,9	2.68	0.12	2,8	0.44	0.66	2,8	1.35	0.31
Male color	1,9	0.05	0.83	1,8	0.01	0.94	1,8	4.39	0.07
Treatment*male color	2,9	2.56	0.13	2,8	0.60	0.57	2,8	0.72	0.52
Laying date	1,8	0.04	0.84	**1,8**	**5.43**	**0.048**	1,7	0.93	0.37

^*^Please be aware that although sample size is not very high (see the number of nest for each analysis above) all nests were monitored in control and experimental conditions and, hence, for each variable the difference between the treatment and the previous time may be useful.

Laying date, which was included as a covariate in all models, was removed from the models when it was far from significance (P > 0.1). Significant terms of the final model are highlighted in bold. Degrees of freedom for fixed effects were estimated using the Kenward-Roger approximation.

## Discussion

We have shown for the first time that, in birds, the extent of melanic plumage coloration may reveal inter-individual differences in the use of social information as inferred by behavioural and fitness-related responses to the exposure to heterospecific calls. Indeed, brownish scops owl females were more likely to respond to social cues than greyish ones. Actually, predation risk at the nests indicated by little owls’ alarm calls only modified fitness of brownish females, which raised fledglings with lower levels of NAbs than greyish ones. In addition, when we analysed parental behaviour in relation to social information treatments, we found that brownish females increased their nest attentiveness in response to the experiment in alarm nests, and that prey delivered to owlets of brownish females were larger during-treatment compared to the pre-treatment time in non-alarm and control nests but not in alarm nests. These results altogether would imply that proneness to rely on social information on predation risk would be related to pheomelanic coloration so that more pheomelanic brownish females would be more sensitive to variation in social cues provided by little owl calls as they disregard little owls’ contact calls, while, in contrast, seem to be stressed in the face of little owls’ alarm calls.

Males bring most of the prey to nests at the beginning of the nesting period (81.8% of prey items)^49^, own unpublished data, and, hence, the effect of the experiment on prey size is likely to be mediated by males. A likely mechanism that could explain changes in male provisioning in relation to female morph might be a response of males to the potential stress suffered by females, since stressed individuals have been shown to be a strong source of stress for their partners^50^. That is to say, potentially stressed brownish females could transmit stress to their partners, which, in response, could show a diminished hunting capacity and, hence, would provide small prey to nests.

The fact that females but not males responded directly to the experiment in relation to their color is intriguing, but might be related to the fact that females remain in the nest during brooding, when the experiment was performed, and hence, are more likely to perceive the threat stimulus than males. In addition, we cannot discard that the lack of effect of the male morph could be due to a low power (Power = 0.18–0.20 in analyses including the male morph) to detect large effect sizes (0.40).

How the social information experiment might induce variation in nestling immunity can potentially be explained by two mutually non-exclusive mechanisms. Firstly, there could be a direct potential stressful effect of the social information manipulation on mothers, who, in response, could change their brooding behaviour (either caring or feeding nestlings). Indeed, immune outcomes in birds have been shown to be strongly determined by these two factors, stress^51^ and food^52^. Our results indicate that brownish females increased their nest attentiveness in response to the experiment in alarm nests, which points toward an effect of our experiment on female level of stress that may affect nestlings’ immunity. However, we did not find a change in maternal provisioning behaviour in response to the experiment that, together with the low provisioning rate of females (see above), suggests that changes in owlet immunity would unlikely be mediated by female provisioning. Second, changes in owlet immunity could arise by the transmission to males of the stress that the social information manipulation could induce on females. In this case, males of brownish females could detect the response of their females to the experiment and modify their parental behaviour accordingly, which could secondarily influence offspring. In agreement with this scenario, the pattern of prey size delivery in the nests is different for nests with greyish and brownish females. In the former, prey size does not change between the during-treatment and pre-treatment periods, whereas in the second, the owlets are fed with larger preys in the during-treatment period (see control group in Fig. 2b). This increase in prey size delivered in non-alarm and control nests of brownish females seems to be a normal pattern in response to foraging conditions that could improve with the progress of the night, because we always recorded parental behaviour first in control and then in experimental conditions (see Methods). Hence, owlets in nests of brownish females, when exposed to the alarm treatment, were not receiving the usual amount of bigger prey provided by males, which may potentially render a lower owlet immunity.

Usually, more melanic individuals are expected to be more aggressive, defensive and exploratory^24,53^. In scops owls, brownish individuals have the highest amounts of phaeomelanin in their feathers^40^ and brownish females are more aggressive than more greyish ones, which would fit well with this expectation. Furthermore, more phaeomelanic barn owls are more prosocial individuals^39^, showing a link between phaeomelanin and sociality in that species. In agreement, here we have found that brownish more-phaeomelanic females were more responsive in the wild to social information than greyish ones. This could indicate that the propensity to use social information might be based on sociability, which could determine the capacity or the easiness to gather social cues. Additionally, color morph could have major importance in other ecological contexts in which social information is involved^54^ as when assessing habitat quality or mate quality. All this could help scops owls to cope with a broad range of environmental conditions, allowing the different morphs to perform well in different conditions.

The fact that only brownish individuals are sensitive to social information could help to understand responses to social cues at the population level. Thereby, analysing the global response of a population to social cues we ignore intraspecific variability in the propensity to use social information, which could lead to contradictory results for instance in different years with annual variation in morph frequency within a breeding population (more or less brown breeders). This emphasizes the idea of explicitly incorporating intraspecific variation into population studies about social information use^18^. As far as we are aware, our study provides a first piece of evidence showing that within-species variation in melanism might covary with the propensity to use social information and, hence, due to this linkage, that the propensity to rely on social cues might be less plastic than expected within a species. Knowing whether the mechanism linking the use and benefits of social information to melanism is general merits further investigation in other taxa as it may greatly contribute to explain the long-term maintenance of melanin-based color polymorphisms in the wild, wherever individuals showing alternative color variants performed differently under variable ecological conditions in which social information is involved^54^.

## Material and Methods

### Study system

The study was performed from 2012 to 2014 in the Hoya of Guadix-Baza, Granada province, southeast of Spain (37°18′N, 3°11′W). The area is an extensive agricultural landscape with scattered holm oaks (*Quercus ilex*) where cork-made nest-boxes were placed to favour the reproduction of a cavity-nesting bird community (for more details see^41^) including scops owls.

The scops owl is a medium-sized, nocturnal and trans-Saharan migrant owl arriving from their winter African quarters to the study area in April^49,55^. In the area, scops owls begin reproduction throughout May^55^, making one clutch per year of about 2–6 eggs. Incubation takes 24–25 days, and is performed by the female. Both sexes participate in feeding tasks through the nestling period^49^. Plumage coloration in the species varies from grey to brown so that individuals can be assigned to one of two distinct color morphs in relation to the amount of phaeomelanin: greyish or brownish.

### Field data collection

Every year, from the beginning of the breeding season, nest-boxes were visited once a week until egg-laying was detected. After detection of a breeding attempt, nests were visited once more just before the estimated hatching date to capture the incubating female by hand during incubation. After hatching, nests were monitored by weekly visits and at the end of the nesting period, we recorded the number of fledglings and fledgling weight. Males were captured at nights by means of nest-traps, when they brought food to offspring. All individuals were ringed with individually numbered metal rings and sexed based on inspection of the brood patch (only present in females^49^). Moreover, upon capture, all adults were photographed for color characterization as in Parejo, *et al*.^56^. In brief, by focusing on redness extension at the head, breast and wings–back, we scored each body part among 1 to 3 points depending on the degree of redness^56^. At the end, scores of the three body parts were summed to get an individual score for every bird ranging from 3 to 9. Individuals were then classified as either brownish (score >7) or greyish (score ≤7).

When the youngest chick of each nest was 12 days old, we extracted blood from nestlings to obtain innate (agglutination activity) immunity measurements. Scops owl chicks hatch asynchronically so that chicks from the same nest may have slightly different ages. Blood was collected from the brachial vein using a 0.5 × 16 mm needle and heparinized capillary tubes to later transfer it into a 1.5 mL tube. Samples were refrigerated immediately after extraction, and later on the same day, plasma and red blood cells were separated by centrifugation at 13300 rpm for 5 min. All samples were stored in a −20 °C freezer until the end of fieldwork when laboratory analyses were performed. Finally, fledglings were weighed to the nearest 1 g using a 300 g Pesola spring balance just before fledging. Ten days later, nests were re-visited to verify fledging success. Nestlings not found dead in the nests during that last visit were considered to have fledged.

### Ethics statement

Animal data collection complies with the current laws of Spain and the fieldwork including adult trapping and obtaining blood samples was authorized by Consejería de Medio Ambiente y Ordenación del Territorio de la Junta de Andalucía (projects CGL2011-27561/BOS and CGL2014-56769-P; licence code: P06-RNM-01862). The study protocol was reviewed and approved by the ethical committee of the Spanish Research Organism (CSIC).

### Laboratory analyses

Assessment of innate humoral immunity was done by using the standard protocol based on Natural Antibody (hereafter NAb) mediated complement activation and red blood cell agglutination^57^.The agglutination responsiveness represents the interaction between NAb and antigens. Quantification of agglutination was done by assessing the dilution stage (on a scale from 1 to 12 titres) at which this reaction stopped against the same amount of rabbit blood cell suspension on digitalized images (for more details on the method see^57,58^). This assay determines the values of circulating NAbs by measuring red blood cell agglutination. We did not consider lytic activity, which can be feasibly determined by this assay, because it did not show variation in nestlings.

### Experimental manipulation of social information on predation risk

Nests with hatchlings were randomly assigned to one of the following treatments: (i) “Alarm” (N = 20 nests), in which we broadcasted little owls’ alarm calls through all the nestling period to simulate threatened little owls; (ii) “Non-alarm” (N = 17 nests), in which we broadcasted non-alarm calls of little owls through all the nestling period to simulate the presence of non-threatened little owls; and (iii) “Control” (N = 14 nests), in which no playback was broadcasted but visits were as frequent as to alarm and non-alarm nests. Therefore, Alarm and Non-Alarm nests are informed nests, while Control nests are uninformed ones.

Neither laying date (General lineal model with a Normal error distribution: treatment effect: F_2,42_ = 0.25, P = 0.78; year effect: F_2,42_ = 19.71, P < 0.0001; treatment * year effect: F_4,42_ = 1.35, P = 0.27), nor clutch size (General linear model with a Poisson error distribution: treatment effect: F_2,42_ = 0.05, P = 0.95, year effect: F_2,42_ = 0.45, P = 0.64; treatment * year effect: F_4,42_ = 0.06, P = 0.99), nor female morph (Logistic regression model: treatment effect: χ_2_ = 1.54, P = 0.46; year effect: χ_2_ = 2.02, P = 0.36; treatment * year effect: χ_4_ = 4.82, P = 0.31) differed among nests assigned to the different treatments, indicating that the experiment was correctly randomized.

In the second and third years of the study, all territories that were reused by scops owls (5 territories) were assigned to one alternative treatment of the previous year to discard the possibility of biased results due to differences in territorial quality. Furthermore, none of the reused territories were consecutively occupied by the same individuals; therefore, a possible effect of the reutilisation of territories by the same individuals in our experiment can be discarded. On the other hand, the 10 females that were breeding in the population in consecutive years were assigned to alternative treatments in the different years to avoid accumulative effects of the experiment. Nevertheless, we acknowledge that the inclusion of some females more than once in the experiment could affect results due to the non-independence of observations of the same female and to carry-over effects. Therefore, we analysed the effects of the treatment on parental fitness twice, first including data from all the recorded reproductive events and, second including females only the first year they were involved in the experiment. Results were qualitatively identical, and hence we reported in the results section those performed on all the reproductive events (including non-repeated and repeated females) and in the Supplementary Material (Table S2) those performed on the limited data set (including non-repeated females).

Call treatments were applied five times to every nest each 3 days from the day all eggs had hatched. Calls were broadcasted close to scops owls’ nests during 2 h each day after dusk. For that purpose, we used portable amplified speakers connected to digital takeMS audio players (model deseo) as in^5,41^. Three non-alarm and three alarm calls from different individuals were used to generate two distinct 1.5–3 min of uncompressed audio files with the recordings of the various calls. These two audio files were randomly mixed with periods of silence from 3 to 8 min and then randomly broadcasted to reduce pseudoreplication. Little owl calls and silent periods were recorded in separate tracks so that the exact sequence of calls and silences was randomly decided by selecting the random playback mode. The randomized presentation of the three calling bouts of each type in combination with the silence tracks during the 2 h of broadcasting produces an unique assortment of calls for each day of treatment and nest (see for similar approaches^5,59,60^). Average broadcasting volume was 89.1 (+1.1) dB measured 1 m away from the speaker, which closely resembles by ear to the natural production of real little owl calls.

### Parental care

In addition, in 2014, coinciding with the second visit to nests for call broadcasting, we recorded parental behaviour by filming nests within the same day before (during 60–90 min just after sunset, which is just after dusk) and after the beginning of the broadcasting (during 60–90 min once the broadcasting had begun). Thus, each nest was the same day first filmed in control conditions (pre-treatment time) and afterwards under experimental conditions (during-treatment time). Therefore, a potential effect of time of day on foraging remains to be controlled for. Infrared mini cameras (KPC- S500, black and white CCD camera, Esentia Systems Inc.) (Size of 25 mm (W) × 25 mm (H)) were used to record parental activity at night. Cameras were installed in the middle of the roofs of unused nestboxes that were interchanged with the roofs of the target nestboxes the filming day prior to monitoring, so that they were not likely to be detected by birds due to their small size. All the other necessary material for recording such as cables, batteries and recording discs and more likely to be detected by birds was camouflaged in the tree supporting the nestbox. Females had been marked in the head using white Tippex before filming, which allowed us to identify parents in video recordings. One observer, who was blind to time (i.e. before or during treatment) and to the treatments assigned to nests (Alarm N = 7 nests, Non-alarm N = 6 nests and Control N = 5 nests), extracted from recordings: (i) Latency as the time elapsed from the onset of filming until each of the parents return to normal activities such as brooding, cleaning or feeding at nests. In the case of males, this latency usually reflects the time elapsed until they resume bringing prey to the nest. However, at the time we recorded parental behaviour, females spend a lot of time inside nest-boxes brooding chicks and they only sometimes leave the nests to hunt. While females are in nests they usually brood nestlings, get and distribute prey brought by males among nestlings. Therefore, we measured latency for females as the time they take on their own to return to normal activities at nests, i.e. brooding, cleaning or feeding. (ii) Parental provisioning rate as the number of prey per hour of the adults separately; and (iii) mean prey size per observation period. The size of prey was estimated by comparing their length to the length of adult’s head, i.e. giving a value of 1 to prey as long as the head of the adult and relativizing the others. The difference between during- and pre-treatment periods in all these variables for a given nest were used as dependent variables in all analyses.

### Statistical analyses

Analyses were performed using SAS v.9.4 statistical software (SAS 2002–2008 Institute, Cary, NC, USA).

We first analysed whether laying date (by performing a General lineal model, GLM procedure in SAS with normal error distribution and identity link function), clutch size (by performing a General lineal model, GLIMMIX procedure in SAS with Poisson error distribution and log link function) and female color (by performing a logistic regression model, GENMOD procedure in SAS) differed among nests assigned to the different treatments, and hence whether the experiment was correctly randomized. In all these models, to take into account environmental effects, the year and the interaction between year and treatment were introduced as fixed factors

Then, we performed General lineal models to test for the effect of the experimental treatment on the number of fledglings per nest (Genmod procedure in SAS with Poisson error distribution, log link function), mean weight of fledglings per nest (GLM procedure in SAS with Normal error distribution, identity link function) and mean NAb levels (GLM procedure in SAS with Normal error distribution, identity link function) per nest. In these models we introduced the treatment (with three levels: alarm, non-alarm and control) and the female color (with two levels: brownish *versus* greyish) as factors, and the interaction treatment × female color to evaluate the expected possibility of changing responses to the experiment with female color. In addition, laying date was introduced in models as a covariate to account for individual quality and the year as a fixed effect to take into account environmental effects. When studying the number of fledglings per nest, only nests that avoided predation were included.

We performed General lineal models (GLM procedure in SAS, Normal distribution, link = identity) with the variables measuring parental care (change in latency, provisioning rate and mean size of provisioned prey from pre-treatment to during-treatment time) as dependent variables in each model, and the treatment, individual color and its interaction as explanatory variables. In all models, laying date was introduced as a covariate to account for individual quality.

Pairwise differences in significant models were checked by comparisons of least-squared means of each treatment.

## Supplementary information

Melanism influences the use of social information in a polymorphic owl.

## Data Availability

The datasets generated during and/or analysed during the current study are available from the corresponding author on reasonable request.
