# Supplementary material for: Melanism influences the use of social information in a polymorphic owl
Source: Sci Rep. 2020 Feb 5;10:1869. doi: 10.1038/s41598-020-58826-6 (PMC7002771; doi:10.1038/s41598-020-58826-6)
Supplement: Supplementary file 1 — Melanism influences the use of social information in a polymorphic owl. [file 41598_2020_58826_MOESM1_ESM.docx]

**Melanism influences the use of social information in a polymorphic owl**

**Deseada Parejo^1,2*^ & Jesús M. Avilés^2^**

^1^Área de Zoología, Departamento de Anatomía, Biología Celular y Zoología, Facultad de Ciencias, Universidad de Extremadura, Badajoz (Spain). E-mail: dparejo@unex.es

^2^Departamento de Ecología Funcional y Evolutiva, Estación Experimental de Zonas Áridas (EEZA-CSIC), Almería (Spain)

**Table S1**. Detailed results of general linear models testing for the effect of the experimental treatment and the individual color on the number of fledglings, mean weight of fledglings and mean NAb levels in fledglings per nest. Laying date, which was included as a covariate in all models, was removed from the models when it was far from significance (P > 0.1). Significant terms of the final model are highlighted in bold. Degrees of freedom for fixed effects in models with Normal error distribution were estimated using the Kenward-Roger approximation.

|  | **Number of fledglings**  n = 43 nests | | | | | **Mean fledgling weight**  n = 40 nests | | | | | **Mean Nab level in fledglings**  n = 42 nests | | | | |
| --- | --- | --- | --- | --- | --- | --- | --- | --- | --- | --- | --- | --- | --- | --- | --- |
|  |  |  |  |  |  |  |  |  |  |  |  |  |  |  |  |
|  | ß ± SE | DF | χ | p | ß ± SE | | DF | F | p | ß ± SE | | DF | F | p |  |
| Intercept | **4.81 ± 1.86** | **1** | **6.67** | **0.01** | **126.39 ± 24.88** | | **31** | **5.08** | **<0.001** | **9.19 ± 1.28** | | **34** | **12.49** | **<0.0001** |  |
| Treatment† | Control: Reference |  |  |  | Control: Reference | |  |  |  | **Control: Reference** | |  |  |  |  |
|  | Alarm: -0.02 ± 0.41 | 2 | 1.49 | 0.47 | Alarm: 1.35 ± 5.64 | | 2,31 | 0.53 | 0.59 | **Alarm: -2.91 ± 0.78** | | **2,34** | **7.13** | **0.003** |  |
|  | Non-alarm: -0.02 ± 0.37 |  |  |  | Non-alarm: 2.81 ± 5.10 | |  |  |  | **Non-alarm: -0.81 ± 0.70** | |  |  |  |  |
| Female color (Greyish)† | Greyish: -0.21 ± 0.37 | 1 | 1.43 | 0.23 | Greyish: 6.11 ± 4.94 | | 1,31 | 0.19 | 0.67 | Greyish: -0.05 ± 0.70 | | 1,34 | 2.84 | 0.10 |  |
| Treatment*female color† | Control*Greyish: Reference |  |  |  | Control* Greyish: Reference | |  |  |  | **Control*** **Greyish: Reference** | |  |  |  |  |
|  | Alarm*Greyish: 0.23 ± 0.51 | 2 | 1.42 | 0.49 | Alarm* Greyish: -3.06 ± 6.99 | | 2,31 | 1.62 | 0.21 | **Alarm* Greyish: 2.21 ± 0.97** | | **2,34** | **3.97** | **0.03** |  |
|  | Non-alarm* Greyish: -0.35 ± 0.50 |  |  |  | Non-alarm* Greyish: -11.60 ± 6.76 | |  |  |  | **Non-alarm* Greyish: -0.10 ± 0.96** | |  |  |  |  |
| Laying date | **-0.03 ± 0.01** | **1** | **4.06** | **0.04** | **-0.43 ± 0.18** | | **1,31** | **5.61** | **0.02** | 0.01 ± 0.03 | | 1,33 | 0.24 | 0.63 |  |
| Year | 2014: Reference | 2 | 2.95 | 0.23 | **2014: Reference** | |  |  |  | **2014: Reference** | |  |  |  |  |
|  | 2013: 0.35 ± 0.25 |  |  |  | **2013: 9.16 ± 3.60** | | **2,31** | **5.78** | **0.01** | **2013: 3.95 ± 0.44** | | **2,34** | **43.91** | **<0.0001** |  |
|  | 2012: -0.01 ± 0.35 |  |  |  | **2012: -1.30 ± 4.36** | |  |  |  | **2012: 1.01 ± 0.47** | |  |  |  |  |

†Reference category for treatment was set to “control treatment”, for individual color to “brownish” and for year to “2014”.

**Table S2**. Detailed results of general linear models testing for the effect of the experimental treatment and the individual color on the number of fledglings, mean weight of fledglings and mean NAb levels in fledglings per nest when we only included nests raised by females the first year they were involved in the experiment. Laying date, which was included as a covariate in all models, was removed from the models when it was far from significance (P > 0.1). Significant terms of the final model are highlighted in bold. Degrees of freedom for fixed effects in models with Normal error distribution were estimated using the Kenward-Roger approximation.

|  | **Number of fledglings**  n = 32 nests | | | | **Mean fledgling weight**  n = 29 nests | | | | | **Mean Nab level in fledglings**  n = 31 nests | | | | |
| --- | --- | --- | --- | --- | --- | --- | --- | --- | --- | --- | --- | --- | --- | --- |
|  |  |  |  |  |  |  |  |  |  |  |  |  |  |  |
|  | ß ± SE | DF | χ | p | ß ± SE | DF | F | p | ß ± SE | | DF | F | p |  |
| Intercept | **1.28 ± 0.33** | **1** | **14.53** | **<0.01** | **145.22 ± 25.82** | **28** | **5.63** | **<0.001** | **7.62 ± 0.64** | | **23** | **11.87** | **<0.0001** |  |
| Treatment† | Control: Reference |  |  |  | Control: Reference |  |  |  | Control: Reference | |  |  |  |  |
|  | Alarm: -0.16 ± 0.54 | 2 | 2.54 | 0.28 | Alarm: 0.24 ± 6.72 | 2,28 | 0.06 | 0.94 | Alarm: -2.97 ± 0.97 | | 2,23 | 3.10 | 0.06 |  |
|  | Non-alarm: -0.18 ± 0.39 |  |  |  | Non-alarm: 3.92 ± 5.04 |  |  |  | Non-alarm: -0.92 ± 0.72 | |  |  |  |  |
| Female color (Greyish)† | Greyish: -0.26 ± 0.39 | 1 | 1.56 | 0.21 | Greyish: 3.56 ± 5.10 | 1,28 | 0.45 | 0.51 | Greyish: -0.53 ± 0.75 | | 1,23 | 1.52 | 0.23 |  |
| Treatment*female color† | Control*Greyish: Reference |  |  |  | Control* Greyish: Reference |  |  |  | **Control*** **Greyish: Reference** | |  |  |  |  |
|  | Alarm*Greyish: 0.25 ± 0.64 | 2 | 1.35 | 0.51 | Alarm* Greyish: 2.47 ± 8.01 | 2,28 | 0.81 | 0.46 | **Alarm* Greyish: 3.12 ± 1.16** | | **2,23** | **4.53** | **0.02** |  |
|  | Non-alarm* Greyish: -0.45 ± 0.55 |  |  |  | Non-alarm* Greyish: -6.62 ± 6.89 |  |  |  | **Non-alarm* Greyish: 0.14 ± 0.99** | |  |  |  |  |
| Laying date | -0.02 ± 0.01 | 1 | 2.30 | 0.13 | **-0.58 ± 0.19** | **1,28** | **9.30** | **0.006** | 0.004 ± 0.03 | | 1,22 | 0.02 | 0.89 |  |
| Year | 2014: Reference | 2 | 2.96 | 0.23 | **2014: Reference** |  |  |  | **2014: Reference** | |  |  |  |  |
|  | 2013: 0.05 ± 0.28 |  |  |  | **2013: 12.80 ± 3.37** | **2,28** | **5.73** | **0.01** | **2013: 4.17 ± 0.54** | | **2,23** | **31.50** | **<0.0001** |  |
|  | 2012: -0.44 ± 0.30 |  |  |  | **2012: 1.87 ± 4.50** |  |  |  | **2012: 0.94 ± 0.48** | |  |  |  |  |

†Reference category for treatment was set to “control treatment”, for individual color to “brownish” and for year to “2014”.

**Table S3**. Detailed results of general lineal models testing the effect of the experimental manipulation and individual color morph on differences between pre- and during-treatment time in latency of males and females to resume usual activities, in provisioning rate of male and females and in mean size of provisioned prey per nest. Laying date, which was included as a covariate in all models, was removed from the models when it was far from significance (P > 0.1). Significant terms of the final model are highlighted in bold. Degrees of freedom for fixed effects were estimated using the Kenward-Roger approximation.

|  | **Females** | | | | | | | | | **Both adults** | | | | | |  |
| --- | --- | --- | --- | --- | --- | --- | --- | --- | --- | --- | --- | --- | --- | --- | --- | --- |
|  | **Latency**  n = 18 nests | | | | **Provisioning rate**  n = 18 nests | | | | **Mean prey size**  n = 17 nests | | | | | |  |  |
|  |  | | | |  |  |  |  |  |  |  |  |  |  |  |  |
|  | ß ± SE | DF | F/*t* | p | ß ± SE | DF | F/*t* | p | | | ß ± SE | DF | F/*t* | p | | |
| Intercept | **11823 ± 2717.18** | **11** | **4.35** | **0.001** | 0.60 ± 1.98 | 12 | 0.30 | 0.76 | | | 0.29 ± 0.15 | 11 | 1.88 | 0.09 | | |
| Treatment† | Control: reference |  |  |  | Control: reference |  |  |  | | | Control: reference |  |  |  | | |
|  | Alarm: -1645.49 ± 774.80 | 2,11 | 1.64 | 0.24 | Alarm: -1.39 ± 2.28 | 2,12 | 0.66 | 0.53 | | | Alarm: -0.47 ± 0.19 | 2,11 | 1.89 | 0.20 | | |
|  | Non-alarm: -81.64 ± 780.90 |  |  |  | Non-alarm: -1.06 ± 2.28 |  |  |  | | | Non-alarm: -0.16 ± 0.18 |  |  |  | | |
| Female morph (Greyish) † | -756.88 ± 717.92 | 1,11 | 0.05 | 0.82 | -1.69 ± 2.21 | 1,12 | 0.39 | 0.55 | | | **-0.42 ± 0.17** | **1,11** | **6.84** | **0.02** | | |
| Treatment*female morph† | **Control*Greyish** |  |  |  | Control*Greyish |  |  |  | | | **Control*Greyish** |  |  |  | | |
|  | **Alarm*Greyish: 2015.96 ± 889.05** | **2,11** | **4.89** | **0.03** | Alarm*Greyish: 2.64 ± 2.68 | 2,12 | 1.28 | 0.31 | | | **Alarm*Greyish: 0.52 ± 0.22** | **2,11** | **3.89** | **0.05** | | |
|  | **Non-alarm*Greyish: 19.23 ± 931.73** |  |  |  | Non-alarm*Greyish: 4.38 ± 2.74 |  |  |  | | | **Non-alarm*Greyish: 0.08 ± 0.21** |  |  |  | | |
| Laying date | **-73.18 ± 18.10** | **1,11** | **16.35** | **0.002** | -0.06 ± 0.06 | 1,11 | 1.05 | 0.33 | | | -0.00 ± 0.005 | 1,10 | 0.48 | 0.50 | | |
|  | **Males** | | | | | | | | | **Both adults** | | | | | |  |
|  | **Latency**  n = 15 nests | | | | **Provisioning rate**  n = 15 nests | | | | **Mean prey size**  n = 14 nests | | | | | |  |  |
|  |  |  |  |  |  |  |  |  |  | | | | | |  |  |
|  | ß ± SE | DF | F/*t* | p | ß ± SE | DF | F/*t* | p | | | ß ± SE | DF | F/*t* | p | | |
| Intercept | **44.56 ± 81.26** | **9** | **2.49** | **0.03** | **94.86 ± 42.21** | **8** | **2.25** | **0.05** | | | 0.96 ± 0.95 | 7 | 1.00 | 0.35 | | |
| Treatment† | Control: reference |  |  |  | Control: reference |  |  |  | | | Control: reference |  |  |  | | |
|  | Alarm: -52.60 ± 19.57 | 2,9 | 2.68 | 0.12 | Alarm: 1.28 ± 10.78 | 2,8 | 0.44 | 0.66 | | | Alarm: -0.06 ± 0.23 | 2,7 | 1.78 | 0.24 | | |
|  | Non-alarm: -35.74 ± 19.57 |  |  |  | Non-alarm: 13.57 ± 12.50 |  |  |  | | | Non-alarm: 0.47 ± 0.27 |  |  |  | | |
| Male morph (Greyish) † | -26.50 ± 15.98 | 1,9 | 0.05 | 0.83 | 6.54 ± 8.92 | 1,8 | 0.01 | 0.94 | | | -0.11 ± 0.19 | 1,7 | 3.96 | 0.09 | | |
| Treatment*male morph† | Control*Greyish |  |  |  | Control*Greyish |  |  |  | | | Control*Greyish |  |  |  | | |
|  | Alarm*Greyish: 52.58 ± 23.70 | 2,9 | 2.56 | 0.13 | Alarm*Greyish: -4.61 ± 13.30 | 2,8 | 0.60 | 0.57 | | | Alarm*Greyish: 0.07 ± 0.29 | 2.7 | 1.17 | 0.36 | | |
|  | Non-alarm*Greyish: 33.64 ± 23.97 |  |  |  | Non-alarm*Greyish: -16.22 ± 14.76 |  |  |  | | | Non-alarm*Greyish: --0.44 ± 0.32 |  |  |  | | |
| Laying date | -0.11 ± 0.54 | 1,8 | 0.04 | 0.84 | **-0.66 ± 0.28** | **1,8** | **5.43** | **0.048** | | | -0.97 ± 0.01 | 1,7 | 0.93 | 0.37 | | |

*Please be aware that although sample size is not very high (see the number of nest for each analysis above) all nests were monitored in control and experimental conditions and, hence, for each variable the difference between the treatment and the previous time may be useful.

†Reference category for treatment was set to “control treatment” and for individual color morph to “brownish”.
